# Supplementary material for: Genomic Characterisation of UFJF_PfDIW6: A Novel Lytic Pseudomonas fluorescens-Phage with Potential for Biocontrol in the Dairy Industry
Source: Viruses. 2022 Mar 17;14(3):629. doi: 10.3390/v14030629 (PMC8951688; doi:10.3390/v14030629)
Supplement: Supplementary file 1 [file viruses-14-00629-s001.zip › Figures S1 and S2.pdf]

viruses

MDPI

Article

Genomic Characterisation of UFJF_PfDIW6: A Novel Lytic *Pseudomonas fluorescens*-Phage with Potential for Biocontrol in the Dairy Industry

Humberto Moreira Hungaro [1,*,†], Pedro Marcus Pereira Vidigal [2,†], Edilane Cristina do Nascimento [1], Felipe Gomes da Costa Oliveira [1], Marco Túlio Pardini Gontijo [3] and Maryoris Elisa Soto Lopez [4,*]

[1] Departamento de Ciências Farmacêuticas, Faculdade de Farmácia, Universidade Federal de Juiz de Fora (UFJF), Juiz de Fora 36036-900, MG, Brazil; edilanemv@outlook.com (E.C.d.N.); lipygomes12@yahoo.com.br (F.G.d.C.O.)

[2] Núcleo de Análise de Biomoléculas (NuBioMol), Campus da UFV, Universidade Federal de Viçosa (UFV), Viçosa 36570-900, MG, Brazil; pedro.vidigal@ufv.br

[3] Departamento de Genética, Evolução, Microbiologia e Imunologia, Instituto de Biologia, Universidade Estadual de Campinas (UNICAMP), Campinas 13083-872, SP, Brazil; m264546@dac.unicamp.br

[4] Departamento de Engenharia de Alimentos, Universidade de Córdoba (UNICORDOBA), Córdoba 230002, Colombia

* Correspondence: humberto.hungaro@farmacia.ufjf.br (H.M.H.); mesoto@correo.unicordoba.edu.co (M.E.S.L.); Tel.: +55-32-2102-3804 (H.M.H.); +57-310-469-02-04 (M.E.S.L.)

† These authors contributed equally to this work.

Supplementary Material:

Figures S1 and S2

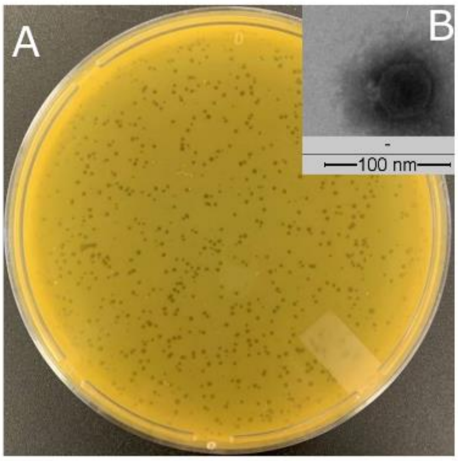

Parameter	Results
Morphology	Icosahedral head (42.8 ± 4.4 nm)<br>Short tail (8.5 ± 0.5 nm)
Titer on host bacterium*	9.7 ± 0.18 log PFU/ml
Lysis plate size*	0.5 ± 0.05 mm
Latent period*	115 min
Burst size*	145 PFU/infected cell
Host-range**	Host specific
Resistance to pH at 25 °C/60 min<br>(titers ranging from)	Stability at pH 3 to 11 for<br>(9.7 to 8.8 log PFU/ml)
Thermal resistance in buffer SM<br>(titer after treatment)	63 °C/30 min (6.9 ± 0.1 log PFU/ml)<br>72 °C/ 30min (4.6 ± 0.6 log PFU/ml)
Resistance to sodium hypochlorite	Inactivated at 50 and 200 mg/l
Effect against *Pseudomonas* in raw milk	Reduction of 3 log CFU/ml

Figure S1. Phenotypic features of *Pseudomonas* phage UFJF_PfDIW6. (**A**) Lysis plaques formed by UFJF_PfDIW6 on the lawn of *P. fluorescens* UFV 041. Incubation conditions: BHI agar at 30 °C for 24 h. (**B**) Virion morphology. Transmission electron micrograph (TEM) negatively stained with 2% uranyl acetate. Bar magnification: 140,000X. *Tests performed in TSB medium at 30 °C; **Test performed against 23 bacterial strains belonging to nine genera. The methodology used for phenotypic characterization of UFJF_PfDIW6 was described in detail by Nascimento et. [27].

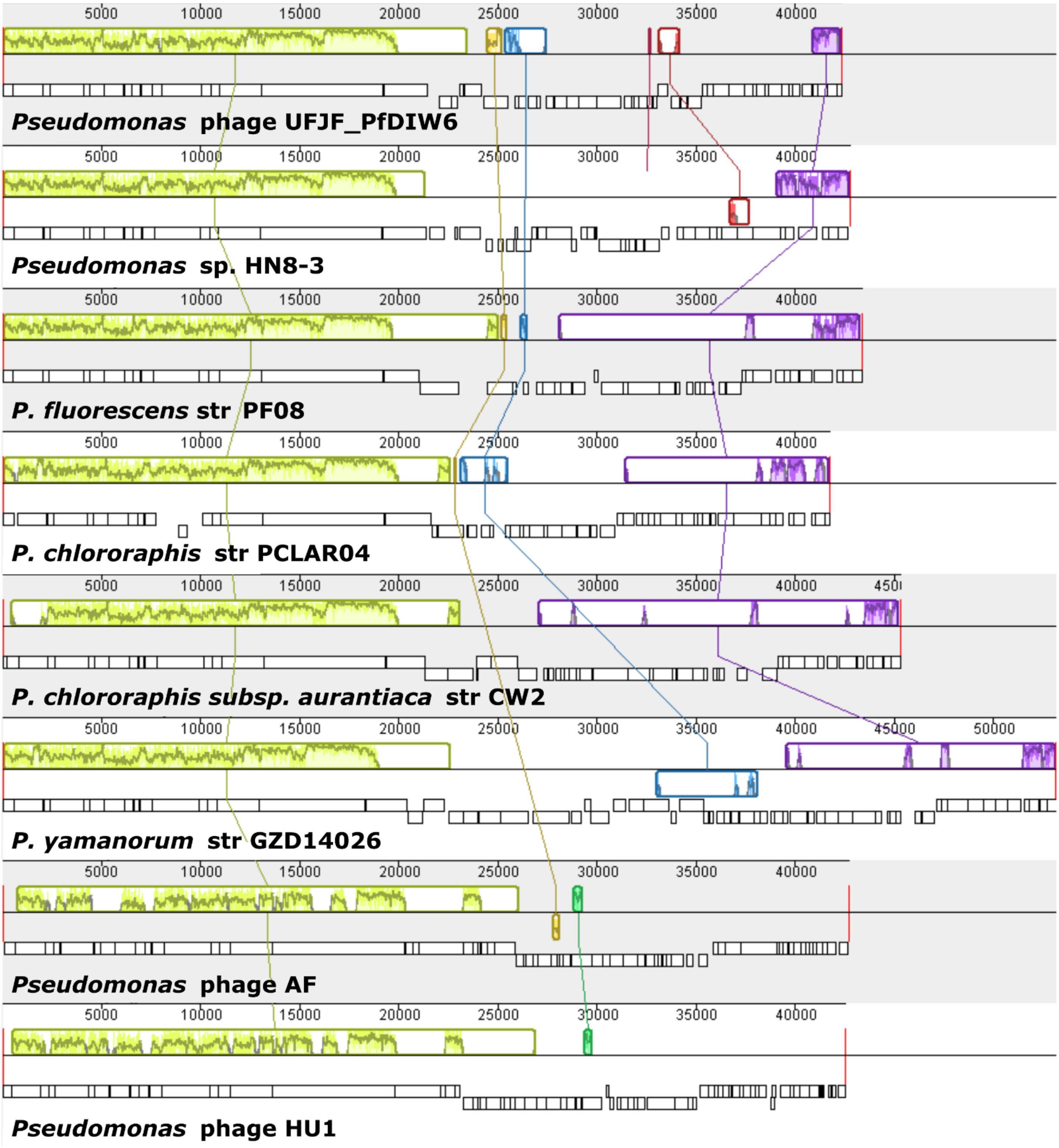

Figure S2. Conserved locally collinear blocks (LCBs) shared among *Pseudomonas* UFJF_PfDIW6 phage, *Pseudomonas* sp. HN8-3 prophage, *P. fluorescens* str PF08 prophage, *P. chlororaphis* str PCLAR04 prophage, *P. chlororaphis* subsp. *aurantiaca* str CW2 prophage, *P. yamanorum* str GZD14026 prophage, Pseudomonas phage AF, and Pseudomonas phage HU1 genomes. The alignment was performed using progressiveMauve with default parameters. The colored blocks correspond to the LCBs predicted by Mauve. Horizontal white bars correspond to the Coding DNA sequences (CDSs) annotated in each genome.
